# A Novel Approach for Cisplatin‐Resistant Esophageal Squamous Cell Carcinoma via Amino Acid Transporter LAT1 Inhibition

**DOI:** 10.1002/cam4.71234

**Published:** 2025-09-09

**Authors:** Takeru Mozumi, Narumi Harada‐Shoji, Yohei Ozawa, Yuto Yamazaki, Ryoyu Niikuni, Kentaro Imai, Yusuke Taniyama, Chiaki Sato, Hiroshi Okamoto, Hirotaka Ishida, Atsushi Kunimitsu, Iku Sasaki‐Higashimoto, Chisa Kobayashi, Shozo Furumoto, Takaaki Abe, Takashi Suzuki, Takashi Kamei

**Affiliations:** ^1^ Department of Surgery Tohoku University Graduate School of Medicine Sendai Japan; ^2^ Department of Breast and Endocrine Surgical Oncology Tohoku University Graduate School of Medicine Sendai Japan; ^3^ Department of Pathology Tohoku University Hospital Sendai Japan; ^4^ Research Center for Accelerator and Radioisotope Science Tohoku University Sendai Japan; ^5^ Division of Nephrology, Endocrinology, and Vascular Medicine Tohoku University Graduate School of Medicine Sendai Japan; ^6^ Department of Medical Science Tohoku University Graduate School of Biomedical Engineering, Tohoku University Sendai Japan; ^7^ Department of Clinical Biology and Hormonal Regulation Tohoku University Graduate School of Medicine Sendai Japan

**Keywords:** cisplatin resistance, esophageal squamous cell carcinoma, JPH203, L‐type amino acid transporter 1, neoadjuvant chemotherapy

## Abstract

**Background:**

Esophageal squamous cell carcinoma (ESCC) represents an aggressive cancer type associated with poor prognosis, often treated with neoadjuvant chemotherapy (NAC) using cisplatin‐based regimens. However, cisplatin resistance limits therapeutic efficacy, necessitating a deeper understanding of resistance mechanisms. L‐type amino acid transporter 1 (LAT1) plays a crucial role in amino acid uptake and is linked to cancer cell survival through activation of the mammalian target of rapamycin (mTOR) pathway. The involvement of LAT1 in cisplatin resistance in ESCC remains unclear.

**Methods:**

LAT1 expression in ESCC patient samples post‐NAC was evaluated by immunohistochemistry, and its association with clinicopathological factors and survival outcomes was analyzed. ESCC cell lines with varying cisplatin sensitivities were assessed for LAT1 expression using western blotting. Amino acid metabolism was examined via radiotracer uptake of ^18^F‐FET and ^18^F‐FDG. RNA sequencing was conducted to identify differentially expressed genes associated with mTOR signaling and autophagy. Finally, the effect of the LAT1 inhibitor JPH203 on cell proliferation was tested.

**Results:**

High LAT1 expression was significantly associated with larger tumor size, lymph node metastasis, advanced pathological stage, and poor NAC response. Patients with high LAT1 expression exhibited shorter disease‐free survival and overall survival. Cisplatin‐resistant ESCC cells (KYSE520) showed elevated LAT1 expression, which further increased following cisplatin treatment. Radiotracer assays revealed that ^18^F‐FET uptake was significantly higher in KYSE520 after cisplatin treatment compared to the sensitive cell line TE5. RNA sequencing identified regulation of mTOR pathway components and autophagy‐related genes in cisplatin‐resistant cells. Treatment with JPH203 significantly suppressed cell proliferation, particularly in KYSE520 cells, indicating LAT1's role in sustaining tumor cell survival under chemotherapy stress.

**Conclusion:**

LAT1 contributes to cisplatin resistance in ESCC by sustaining amino acid metabolism and promoting mTOR‐dependent autophagy. Targeting LAT1 with JPH203 enhances cisplatin sensitivity, suggesting that LAT1 inhibition could be a promising therapeutic strategy for overcoming chemoresistance in ESCC.

## 1 Introduction

Esophageal cancer has a poor prognosis and a significant potential for metastasis since it is a very aggressive malignancy [1]. In Japan, squamous cell carcinoma is the most common, and the standard treatment for locally advanced esophageal squamous cell carcinoma (ESCC) is radical resection with lymph node dissection following neoadjuvant chemotherapy (NAC) [2]. NAC is a preoperative treatment strategy used to shrink tumors before surgery to improve surgical outcomes and reduce the risk of local or distant metastasis [3]. Platinum‐based regimens, including cisplatin, are commonly used for NAC in ESCC [4, 5]. In the currently implemented NAC for ESCC, the response rate is approximately 40%, and the prognosis is poor for the non‐responders to NAC [6]. The variable efficacy of NAC underscores the need to investigate the mechanisms of chemoresistance in esophageal cancer. Various factors contribute to chemoresistance in esophageal cancer [6], but the mechanisms underlying chemoresistance in ESCC remain unclear.

Amino acid metabolism plays crucial roles in cancer development and progression [6]. Cancer cells rely on high nutrient intake, including amino acids, to support their rapid growth, energy needs, and proliferation. To meet these needs, they often upregulate amino acid transporters on their cell membranes, facilitating the increased uptake of essential amino acids (EAA) from the extracellular environment. Additionally, amino acids can contribute to chemoresistance [7, 8]. Cancer cells reprogram their amino acid metabolism pathways and acquire distinct metabolic adaptations to facilitate biosynthetic processes that enable them to counteract the effects of chemotherapy [9, 10]. This metabolic flexibility allows cancer cells to survive and thrive, even under the selective pressure of chemotherapeutic agents.

l‐type amino acid transporter 1 (LAT1) belongs to the solute carrier family of transporters and functions as a transporter of large neutral amino acids such as leucine [11], which is essential for protein synthesis and other metabolic processes. LAT1 is widely expressed in various tissues, including the brain, placenta, and testes [12, 13]. LAT1 is highly expressed in various cancers, where it contributes to tumor growth, metabolism, and survival by providing the essential nutrients required for cell proliferation [14, 15]. LAT1 supplies cancer cells with EAAs necessary for protein synthesis and promotes cancer growth through the activation of the mammalian target of rapamycin (mTOR) pathway [16]. The mTOR pathway is a key regulator of cell growth and proliferation, and its activation by amino acids such as leucine supports cancer cell survival and resistance to therapy [17]. Consequently, elevated LAT1 expression correlates with poor prognosis for several cancers. This overexpression serves as an indicator of an unfavorable prognosis and contributes directly to the development of chemotherapy resistance in cancer. By ensuring a continuous supply of amino acids, LAT1 allows cancer cells to cope with the toxic effects of chemotherapy. Consequently, the inhibition of LAT1 may prevent the uptake of EAAs, which are essential for the proliferation and viability of cancer cells [18, 19]. The selective LAT1 inhibitor JPH203 [(S)‐2‐amino‐3‐(4‐((5‐amino‐2‐phenylbenzo [d] oxazol‐7‐yl) methoxy)‐3, 5‐dichlorophenyl) propanoic acid] has been reported to exert antitumor effects in various cancers [7, 20, 21]. Recent studies have demonstrated that LAT1 is significantly overexpressed in ESCC and is associated with a poor prognosis [22], and competitive inhibition of LAT1 has been shown to suppress cell proliferation and delay tumor growth in ESCC, suggesting that targeting LAT1 could be a viable therapeutic strategy.

In this study, we aimed to clarify: (1) the pathological and clinical relevance of LAT1 in ESCC post NAC; (2) the relationships between LAT1 and chemoresistance in ESCC; and (3) the potential of LAT1 as a novel therapeutic target. By investigating these characteristics, we hope to uncover new insights into the role of LAT1 in ESCC and explore potential avenues for improving treatment outcomes in patients with this challenging cancer type.

## 2 Material and Methods

### 2.1 Patient Cohort and Sample Selection

We obtained clinical data from 98 patients with ESCC who underwent NAC followed by surgical resection with regional lymph node dissection at Tohoku University Hospital in Sendai, Japan, between 2010 and 2018. The administration of NAC adhered to the Japanese Clinical Oncology Group 9907 (JCOG9907) protocol. The tumors were pathologically categorized based on the 8th edition of the Union for International Cancer Control TNM staging classification for esophageal carcinoma. The histopathological classification of the effects of NAC was established as follows: Grade 0: ineffective (no cytological or histological therapeutic effects observed in the primary lesion); Grade 1: slightly effective (Grade 1a, necrosis, fibrosis, or granulomatous changes observed in less than one‐third of the residual tumor; Grade 1b, in one‐third to two‐thirds of the lesion); Grade 2: moderately effective (necrosis, fibrosis, or granulomatous changes observed in more than two‐thirds of the lesion, although viable residual tumor cells were histologically identified); Grade 3: markedly effective (no viable residual tumor cells present) [23].

### 2.2 Neoadjuvant Chemotherapy and Surgery

Preoperative NAC, conducted in accordance with the JCOG 9907 protocol, involved the administration of intravenous cisplatin (80 mg/m^2^) on Days 1 and 22, alongside a continuous intravenous infusion of 5‐fluorouracil (800 mg/m^2^/day) for 24 h on days 1–5 and 22–26 [5]. Thoracoscopic esophagectomy, gastric tube reconstruction, and cervical esophagogastric anastomosis were subsequently performed with regional lymph node dissection [24].

### 2.3 Immunohistochemistry

Immunohistochemical studies were conducted on paraffin‐embedded tissues that were 3 μm thick and 10% formalin‐fixed. The slides were placed in a sterilizer with citrate buffer that was set to 121°C and incubated for 5 min. After that, tissue slices were put in a blocking solution with 10% rabbit serum and left at 25°C for 1 h. Then, they were put in a solution with a LAT1 mouse monoclonal antibody (1:100, KE023 Trans Genics, Hyogo, Japan) and left overnight at 4°C. For the streptavidin‐biotin amplification method, a Histofine Kit from Nichirei Bioscience in Tokyo, Japan, was used. A 3,3′‐diaminobenzidine (DAB) solution (1 mM DAB, 50 mM Tris–HCl water (pH 7.6), and 0.006% H_2_O_2_) was used to confirm the antigen–antibody complex. Hematoxylin was used as a counterstain. Placental and skin cells from humans were used as positive and negative standards, respectively. Immunohistochemical staining was evaluated using the H‐score method with some modifications. An intensity score (0: no membranous staining, 1: low intensity, 2: weak to moderate intensity, 3: high intensity) multiplied by the positive ratio per tumor area from 0 to 10 (0 = 0%; 1 = 1%–10%; 2 = 11%–20%; 3 = 21%–30%; 4 = 31%–40%; 5 = 41%–50%; 6 = 51%–60%; 7 = 61%–70%; 8 = 71%–80%; 9 = 81%–90%; and 10 = 91%–100%), resulting in a score from 0 to 30 [24, 25]. Two independent investigators evaluated the immunohistochemical staining, and the average score was used for analysis to minimize inter‐observer variability. LAT1 total score of ≥ 11 was considered positive. The cutoff values for positivity were defined by the receiver operating characteristic (ROC) curve of the histological response. This immunohistochemical analysis was conducted retrospectively using archived specimens obtained at the time of surgery and stored in the pathology archives of Tohoku University Hospital.

### 2.4 Cell Lines and Culture

ESCC cell lines (TE5, KYSE150, KYSE220, and KYSE520) were cultured in a 1:1 mixture of RPMI‐1640 and Ham's F12 (FUJIFILM Wako Pure Chemical Corporation, Osaka, Japan) with 10% fetal bovine serum (Cosmobio, Tokyo, Japan) and 100 μg/mL penicillin/streptomycin (Thermo Fisher Scientific, MA, USA). Cells were incubated at 37°C in 5% CO_2_. Total and viable cell counts were determined using Trypan Blue staining (Bio‐Rad; #1450021) and subsequently analyzed using the TC10 Automated Cell Counter system (Bio‐Rad; #1450010).

### 2.5 Cell Proliferation Assay

ESCC cells were counted using Cell Counting Kit‐8 (Dojindo, Kumamoto, Japan). ESCC cells (TE5, KYSE150, KYSE220, and KYSE520) were seeded into 96‐well plates. After 24 h, the medium was changed to one with or without cisplatin (AdipoGen Life Sciences, CA, USA) and JPH203 (Cayman Chemical, MI, USA). Vehicle volumes were equivalent in each well. After 48 h or 72 h, the cell viability assay was performed by adding WST‐8 reagent, which contains a highly water‐soluble tetrazolium salt, to each well, and the microplates were incubated for 2 h at 37°C. The absorbance was then measured at 450 nm [25].

### 2.6 Western Blot Analysis

Cells were seeded at 5 × 10^5^ cells/dish in 100 mm dishes containing 10 mL of medium and cultured for 48 h. Then, the medium was switched to one with or without cisplatin, and the cells were incubated for 24 h. The cisplatin concentration was set according to the reference IC50 values for each sample. Protein analysis was performed using a Simple Western System. The antibodies used for protein detection were LAT1 (1:50; Cell Signaling Technology Japan, K.K., Tokyo, Japan) and an anti‐rabbit secondary antibody (proteinsimple, # 042–206). The corresponding band areas of the targets were normalized by those of β‐actin (1:250, Abcam, Cambridge, UK). Bands were visualized and analyzed using the Compass for Simple Western software version 6.1.0 (proteinsimple).

### 2.7 Cellular Uptake of Radiotracer

The fluoroethyl‐L‐tyrosine (^18^F‐FET) used in this experiment was synthesized in‐house according to a previously described method [26]. A total of 5.0 × 10^4^ ESCC cells (TE5 and KYSE520) were seeded per well in 24‐well plates for the cellular uptake assay. After 24 h, the medium was replaced with one that contained either cisplatin (TE5, 2 μM; KYSE520, 20 μM) or did not, and the cells were incubated for 48 h. The medium was changed to DMEM without glucose (FUJIFILM Wako Pure Chemical Corporation, Osaka, Japan) with fluorodeoxyglucose F 18 (^18^F‐FDG) (Nihon Medi‐Physics, Tokyo, Japan) or ^18^F‐FET (2 μCi/well) and incubated for 1 h. After that, the cells were washed with phosphate buffered saline, lysed, and suspended in 0.5 M sodium hydroxide solution and 0.5 M hydrochloric acid. Radioactivity was measured using a γ‐counter (AccuFLEX γ7000; Hitachi Aloka Medical, Tokyo, Japan). The total protein concentration of the samples was determined using a protein assay kit (Qubit; Thermo Fisher Scientific, Tokyo, Japan).

### 2.8 RNA‐Sequencing and Data Analysis

TE5 and KYSE520 cells were cultured and exposed to drug‐containing medium as described in the western blot analysis section, followed by trypsinization to collect the cells. The drugs used were cisplatin and JPH203, with cisplatin concentrations varied as for the western blot analysis, and JPH203 used at a concentration of 100 μM. After cell collection, total RNA was extracted using an RNeasy Mini Kit (QIAGEN, Hilden, Germany). RNA concentration was measured using a Qubit 4 Fluorometer (Thermo Fisher Scientific, USA), and its quality was assessed using an Agilent 4150 TapeStation System (Agilent Technologies). RNA sequencing (RNA‐seq) libraries were prepared using the QIAseq Stranded RNA Library Kit (QIAGEN, Hilden, Germany). Library quality was evaluated using the Agilent 4150 TapeStation System. Sequencing was outsourced to Haplo Pharma (Sendai, Japan), and paired‐end sequencing was performed on an Illumina NovaSeq X platform (Illumina, USA) with a read length of 150 bp. The quality of raw RNA sequence data was assessed using FastQC (v0.11.9), and adapter sequences and low‐quality reads were removed using fastp (v0.20.1). Clean reads were mapped to the GRCh38 reference genome using STAR (v2.7.10a). Gene‐level read counts were quantified using RSEM (v1.3.3). Differential gene expression analysis was performed using DESeq2 (v1.30.1) and edgeR (v3.32.1) with the false discovery rate (FDR) threshold set at < 0.05. The results were visualized using volcano plots and heat maps.

### 2.9 Statistical Analysis

Statistical analyses were conducted using JMP Pro 17 software (SAS Institute, Cary, NC, USA) and GraphPad Prism version 10. Survival differences were assessed using the Kaplan–Meier method and log‐rank test. The disease‐free survival (DFS) was defined as the time between surgery and recurrence. The overall survival (OS) was defined as the time from surgery to death from any cause. Statistical significance was determined using Student's *t*‐test. Statistical significance was set at *p* < 0.05 for all *p* values.

## 3 Results

### 3.1 LAT1 Expression Was Negatively Associated With Histopathological Tumor Regression Grade in ESCC

**FIGURE 1:**
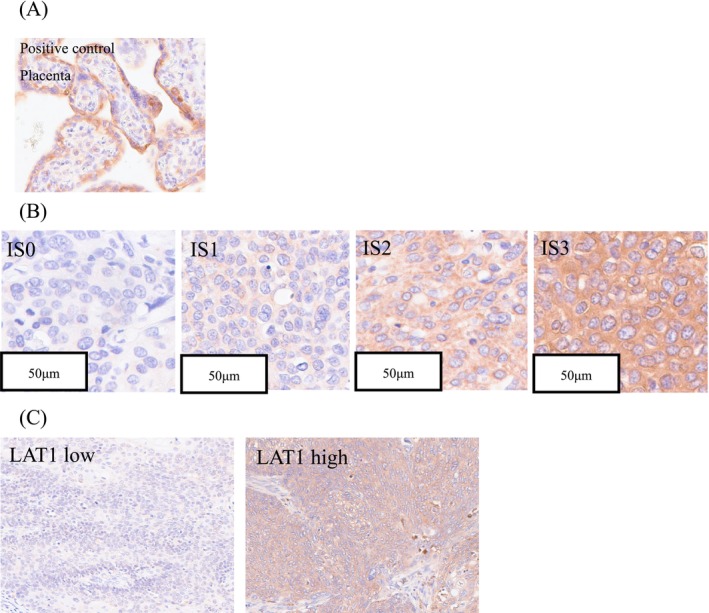
Immunohistochemistry for LAT1 in patients with ESCC receiving neoadjuvant chemotherapy. (A) Immunohistochemistry for LAT1 was performed using human placental tissue as the positive control. (B) Staining intensity was scored on a scale of 0 to 3, where 0 indicated no staining, 1 low intensity, 2 weak to moderate intensity, and 3 high‐intensity staining. (C) LAT1‐low and LAT1‐high tumors were associated with favorable and poor responses to NAC, respectively. Positive LAT1 immunoreactivity was observed in both the cytoplasm and membranes of ESCC cells. A total score of ≥ 11 was considered positive, with the cutoff determined by the ROC curve of the Pathological Therapeutic Grade.

The clinicopathological characteristics of the patients are summarized in Table 1. The results of the correlation analysis of clinicopathological variables and LAT1 immunoreactivity in carcinoma cells are summarized in Table 2. Figure 1A–C show representative microscopic images of LAT1 immunohistochemistry, illustrating the staining patterns observed in the tissue samples.

**TABLE 1:** Clinicopathological factors of ESCC patients with chemotherapy (*n* = 98).

Factors	Number	Percentage (%)
Mean age, (years) (range)	67 (40–84)	
**Gender**		
Male/Female	81/17	82.7/17.3
**Smoking history**		
Yes/No	80/18	81.6/18.4
**Alcohol consumption**		
Yes/No	86/12	87.8/12.2
**pT** ^**a**^		
1/2/3/4	29/19/48/2	29.6/19.4/49.0/2.0
**pN** ^**a**^		
N0/1/2/3	41/28/20/9	41.8/28.6/20.4/9.2
**pStage** ^**a**^		
I/II/III/IV	18/28/40/12	18.4/28.6/40.8/12.2
**Tumor differentiation** ^**b**^		
Unclassifiable/Well/Moderate/Poor	9/23/57/9	9.2/23.5/58.2/9.2
**Lymphatic invasion** ^**b**^		
Ly0/1a/1b/1c	49/25/17/7	50/25.5/17.3/7.1
**Vessel invasion** ^**b**^		
V0/1a/1b/1c	38/27/31/2	38.8/27.6/31.6/2
**Histological NAC efficacy** ^**b**^		
Grade 0/1a/1b/2/3	4/48/24/22/0	4.1/49/24.5/22.4/0

Abbreviation: NAC‐neoadjuvant chemotherapy.

^a^ Tumor‐node‐metastasis (TNM) classification based on the 8th edition of the TNM classification of malignant tumor.

^b^ Histopatohogical features based on the Japanese Classification of Esophageal Cancer, 12th edition (Japan Esophageal Society 2022).

**TABLE 2:** Clinicopathological factors with the status of LAT1 (*n* = 98).

Variables	*N*	LAT1‐Low	LAT1‐High	*p*
**Age (years)**				0.3083
≧ 67	53	23	30	
< 67	45	15	30	
**Sex**				0.6553
Male	82	31	51	
Female	16	7	9	
**Smoking**				0.9133
Yes	80	31	49	
No	18	7	11	
**Alcohol**				0.8263
Yes	86	30	56	
No	12	4	8	
**pT**				0.0022^a^
pT1‐2	48	26	22	
pT3‐4	50	12	38	
**pN**				0.0007^a^
pN0	41	24	17	
pN1‐3	57	14	43	
**pStage**				0.0049^a^
pStage I–II	47	25	22	
pStage III–IV	51	13	38	
**Lymphatic invasion**				0.0009^a^
Ly 0	49	27	22	
Ly 1a‐1c	49	11	38	
**Vessel invasion**				0.0020^a^
V 0	38	22	16	
V 1a–1c	60	16	44	
**Histological NAC efficacy**				0.0013^a^
Grade 0–1b	76	23	53	
Grade 2	22	15	7	

Abbreviation: LAT1, L‐type amino acid transporter‐1, SLC7A5, solute carrier family 7 member5.

^a^ Statistical significance.

In surgically resected specimens following NAC, LAT1 expression was significantly correlated with tumor size (*p* = 0.0022), lymph node metastasis (*p* = 0.0007), pathological stage (*p* = 0.0049), lymphatic invasion (*p* = 0.0009), and vessel invasion (*p* = 0.0020). Additionally, high LAT1 expression was significantly associated with the efficacy of histological NAC (*p* = 0.0013).

### 3.2 LAT1 Expression Was Associated With Overall Survival and Disease‐Free Survival in ESCC Who Received NAC

**FIGURE 2:**
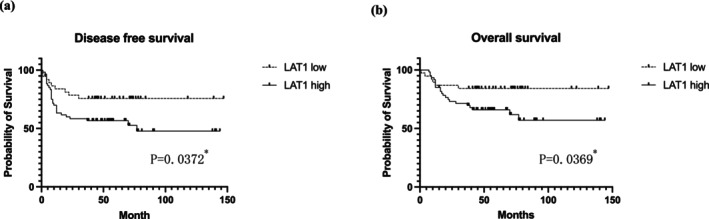
Postoperative survival of patients with ESCC classified according to LAT1 expression. Kaplan–Meier survival curves were used to show survival outcomes, and the log‐rank test was used to assess statistical significance. The analysis revealed that patients with high LAT1 expression had worse DFS (a) and OS (b) than those with low LAT1 expression.

Kaplan–Meier survival analysis revealed that patients with high LAT1 expression levels experienced significantly shorter disease‐free survival (DFS) (*p* = 0.0372, Figure 2a) and overall survival (OS) (*p* = 0.0369, Figure 2b) than those with low LAT1 expression. These findings suggest that LAT1 expression may serve as a prognostic marker of poor outcomes in ESCC.

### 3.3 Evaluation of Cisplatin Sensitivity in ESCC Cell Lines Using Cell Proliferation Assay

**FIGURE 3:**
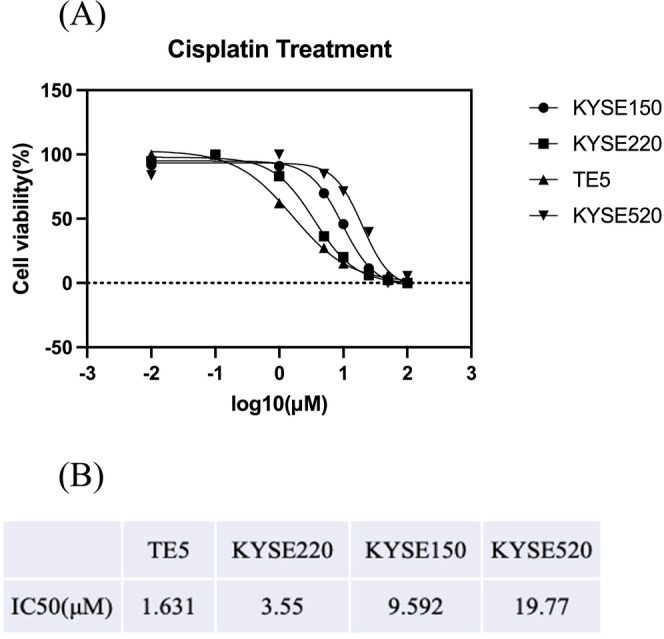
Cell proliferation assays using cisplatin on four ESCC cell lines. (A) Effect of cisplatin on cell proliferation of ESCC cell lines, KYSE150, KYSE220, KYSE520, and TE5 at 48 h. (B) Summarization of the IC50.

We performed cell proliferation assays to evaluate the proliferation of four ESCC cell lines (TE5, KYSE150, KYSE220, and KYSE520) treated with cisplatin. The cells exhibited varying levels of sensitivity to cisplatin, with TE5, KYSE220, KYSE150, and KYSE520 showing IC50 values of 1.631, 3.55, 9.592, and 19.77 μM, respectively (Figure 3A,B). TE5 was the most sensitive, whereas KYSE520 was the most resistant to cisplatin.

### 3.4 ESCC Cell Lines Resistant to Cisplatin Expressed Higher Levels of LAT1

**FIGURE 4:**
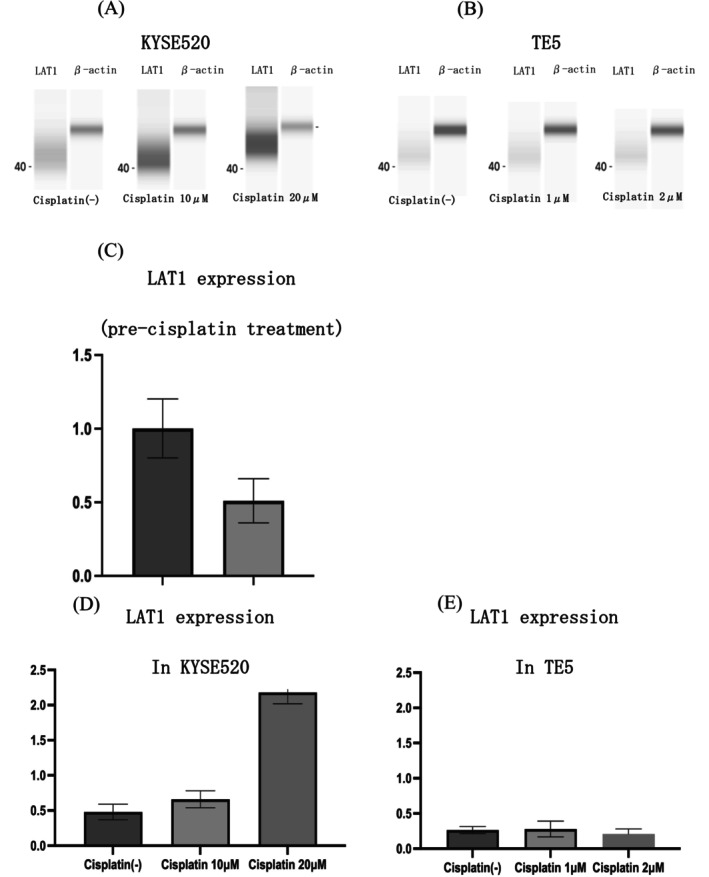
LAT1 expression in KYSE520 (A) and TE5 (B) was confirmed by western blotting and its relative quantity (C). Changes in LAT1 expression after cisplatin treatment in KYSE520 (D) and TE5 (E).

We used western blotting to confirm the expression of LAT1 protein in TE5 and KYSE520 cells and measured LAT1 protein levels in these cells before and after cisplatin treatment. Comparative analysis of TE5 and KYSE520 cells revealed that LAT1 expression was higher in KYSE520 prior to cisplatin treatment (Figure 4A–C). Furthermore, the expression level of LAT1 increased in KYSE520 cells in response to cisplatin treatment in a dose‐dependent manner, while it remained unaltered in TE5 cells (Figure 4A–C). This suggests that LAT1 expression is more strongly induced by cisplatin in cisplatin‐resistant cell lines than in cisplatin‐sensitive cell lines.

### 3.5 Amino Acid Transport via LAT1 Is Relatively Higher in Cisplatin‐Resistant ESCC Cell Line After Cisplatin Treatment

**FIGURE 5:**
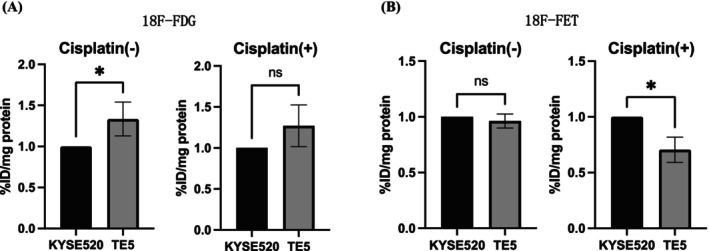
Cellular uptake of radiotracer. (A, B) Cellular uptake of ^18^F‐FDG and ^18^F‐FET in KYSE520 and TE5 with or without cisplatin (20 μM in KYSE520, 2 μM in TE5). *p < 0.05.

To investigate the potential impact of glucose and amino acid metabolism on ESCC cells (TE5 and KYSE520), we evaluated the uptake of ^18^F‐FDG and ^18^F‐FET after cisplatin administration. The uptake of ^18^F‐FDG tended to be higher in TE5 cells than in KYSE520 cells both before and after cisplatin administration (Figure 5A). In contrast, the uptake of ^18^F‐FET showed no difference between TE5 and KYSE520 cells before cisplatin administration; however, after cisplatin administration, the uptake was lower in TE5 than in KYSE520 cells (Figure 5B).

### 3.6 Upregulation of Genes Involved in the mTOR Pathway and Autophagy Observed in Cisplatin‐Resistant ESCC Cell Line

**FIGURE 6:**
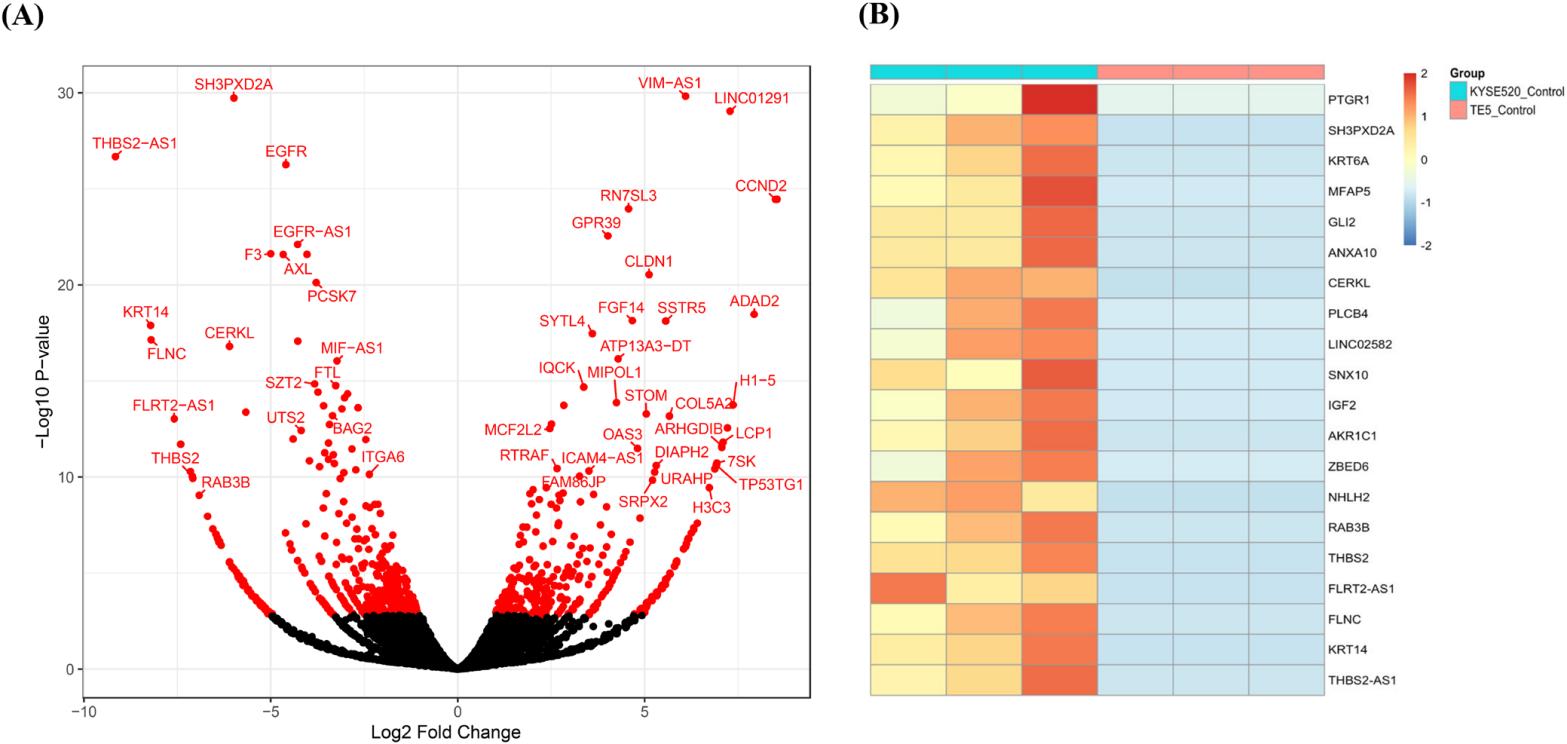
RNA sequencing. (A, B) RNA‐seq results of KYSE520 and TE5 cells in volcano plot and heat map of the top 20 transcripts upregulated in KYSE520 compared to TE5. Differentially expressed genes were identified using a False Discovery Rate (FDR) threshold of < 0.05.

To elucidate the mechanism underlying cisplatin resistance in KYSE520 cells, we performed RNA‐seq of KYSE520 and TE5 cells. The enhanced volcano plots revealed differences in gene expression between the cell lines (Figure 6A). In KYSE520 cells, the expression of multiple genes involved in the mTOR pathway was upregulated. The heatmap also showed that the top 20 transcripts were upregulated in KYSE520 cells compared with those in TE5 cells (Figure 6B). We focused on the genes that were significantly upregulated in KYSE520 cells. The SH3 and PX domains 2A (*SH3PXD2A*) gene, which is closely linked to mTOR signaling, was upregulated in KYSE520 cells. SH3PXD2A acts as a scaffolding protein that interacts with key regulators such as mTOR and unc‐51 like autophagy activating kinase 1 (ULK1), which are crucial for autophagy regulation [27]. This protein is phosphorylated by ULK1, leading to its stabilization and greater involvement in autophagic processes. Autophagy has been shown to play a role in cancer cell survival under stressful conditions such as chemotherapy. Autophagy allows the cells to break down and recycle intracellular proteins, thereby generating amino acids.

In cisplatin‐resistant cells, enhanced autophagy allows for the recycling of intracellular components, maintaining homeostasis and cell viability. SH3PXD2A's involvement in both autophagy and cell migration facilitated by mTOR signaling may contribute to the survival and invasive potential of cisplatin‐resistant KYSE520 cells. In addition, the enhanced amino acid supply via LAT1 provides a constant supply of essential nutrients that fuel autophagy and promote cell survival during cisplatin treatment.

### 3.7 JPH203 Shows Comparable Cell Growth Inhibition in Cisplatin‐Resistant ESCC Cell Line

**FIGURE 7:**
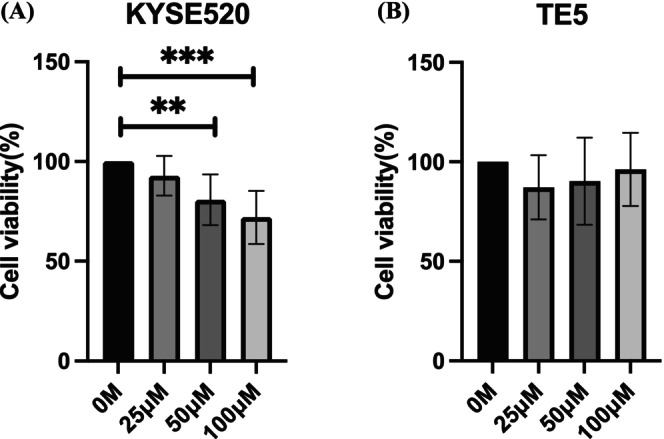
Cell proliferation assays using JPH203 on four ESCC cell lines. (A, B) Cell proliferation assay using JPH203 on the ESCC cell lines KYSE520 and TE5 at 72 h. JPH203 showed comparable cell growth inhibition in KYSE520 which is more resistant to cisplatin. *p* values were evaluated by Student's t‐test. **p < 0.01; ***p < 0.001.

We evaluated the effects of JPH203 on TE5 and KYSE520 cells. JPH203 demonstrated the most potent inhibitory effect on cell proliferation in KYSE520 cells, which are the most cisplatin‐resistant cell line, in comparison to TE5, the most cisplatin‐sensitive cell line (Figure 7A,B). This suggests that JPH203 may be particularly effective against cisplatin‐resistant ESCC cells, such as KYSE520.

## 4 Discussion

In Japan, NAC is the standard treatment for locally advanced ESCC. Currently, NAC for ESCC primarily employs doublet (cisplatin and fluorouracil) and triplet (docetaxel, cisplatin, and fluorouracil) therapies. Triplet therapy demonstrated superior OS compared to doublet therapy in patients with locally advanced ESCC [28]. However, there were more cases of treatment‐related adverse events that led to the discontinuation of NAC in the triplet therapy group. Thus, it is important to select chemotherapy regimens that are tailored for patients from the perspectives of tolerability and efficacy.

Cisplatin is widely used to treat solid tumors through targeting various cellular mechanisms. The primary mechanism involves DNA damage and an increase in oxidative stress [29]. However, cisplatin resistance is a complex mechanism involving several molecular pathways [30, 31, 32]. In a recent study, increased LAT1‐mediated amino acid transport was observed in lung cancer, indicating metabolic reprogramming during tumor development [33]. LAT1 is responsible for the uptake of various EAAs [11] and facilitates the uptake of EAAs and regulates their metabolism, activating pathways such as mTOR [12, 16]. Leucine specifically regulates cellular activities, including apoptosis, and promotes cell proliferation via mTOR [34]. Moreover, LAT1 functions as an antiporter in the glutamate/cysteine (xCT) antiporter system [35], critical for cystine uptake and glutathione synthesis, supporting tumor growth and chemoresistance by maintaining antioxidants and reactive oxygen species (ROS) balance [31, 36]. Thus, LAT1 is pivotal for tumor survival, growth, and chemotherapy resistance.

To our knowledge, this is the first study to demonstrate a correlation between LAT1 and ESCC from the perspective of chemoresistance. Since cisplatin is one of the key drugs commonly used in combination therapy in NAC for ESCC, this study was designed to elucidate the mechanisms associated with cisplatin resistance.

Initially, we collected ESCC patient specimens and clinical data after NAC treatment for immunohistochemical analysis. The results showed a significant correlation between LAT1 status and various clinicopathological factors, as well as poor prognosis in patients with ESCC who underwent NAC. It has been previously demonstrated that the expression of LAT1 is significantly correlated with the prognosis in patients with ESCC who do not receive NAC [22]. We obtained similar results in patients who received NAC in our study. In addition, our findings indicate a correlation between LAT1 status and NAC efficacy in ESCC. A relationship between LAT1 status and chemoresistance has been reported in breast cancer [8], pancreatic ductal adenocarcinoma [37], non‐small cell lung cancer [38], and other cancers. Nevertheless, the correlation between LAT1 status and the therapeutic efficacy of NAC in ESCC has not been previously reported, making this a novel finding.

Next, we investigated how LAT1 status differs between cisplatin‐resistant and ‐sensitive cells and found that LAT1 expression was higher in cisplatin‐resistant cell lines. Furthermore, we observed an increase in LAT1 levels in cisplatin‐resistant cells after cisplatin treatment, which remained unchanged in cisplatin‐sensitive cells. To date, no studies on ESCC have compared LAT1 expression between cisplatin‐resistant and ‐sensitive cell lines, nor have changes in LAT1 expression after cisplatin treatment been reported. Consequently, our study is the first to address this topic. Cisplatin‐resistant cells exhibit higher amino acid levels after cisplatin treatment, with a greater relative increase in LAT1‐transported amino acids compared to cisplatin‐sensitive cells [39]. Amino acid metabolism is regulated by cellular uptake, mTOR‐mediated signaling, and autophagy‐mediated protein degradation [40, 41, 42]. mTOR plays a critical role in regulating autophagy in response to cellular nutrient availability, particularly amino acids [43]. When amino acids are abundant, mTOR activation suppresses autophagy and promotes protein synthesis and cell growth. Conversely, under nutrient‐deprived or stressed conditions, mTOR inhibition induces autophagy, allowing cells to recycle intracellular components, including amino acids, for survival. This balance between mTOR signaling, amino acid metabolism, and autophagy is especially important in cisplatin‐resistant cells, where it may support metabolic adaptations and contribute to drug resistance. Previous studies demonstrated that cisplatin induces autophagy in cancer cells, enabling cisplatin‐resistant cells to recycle intracellular proteins and maintain amino acid homeostasis [44]. Given that LAT1 plays a crucial role in the mTOR pathway, it is likely that LAT1 facilitates the uptake of essential amino acids, thereby contributing to the metabolic adaptations observed in cisplatin‐resistant cells. Additionally, we used a radiotracer to compare the amino acid and glucose metabolism before and after cisplatin treatment. ^18^F‐FDG uptake is associated with glucose metabolism, whereas ^18^F‐FET is associated with amino acid metabolism [45]. Following cisplatin treatment, no relative difference in ^18^F‐FDG uptake was observed between cisplatin‐resistant and ‐sensitive cell lines. However, a change in ^18^F‐FET uptake was observed. While ^18^F‐FET uptake is primarily associated with LAT1 activity, it does not consistently reflect LAT1‐mediated amino acid metabolism, particularly in stress conditions like chemotherapy. In cisplatin‐resistant cancer cells, stress induced by cisplatin treatment resulted in an increased uptake of ^18^F‐FET. This observation supports the hypothesis that chemotherapy‐induced stress stimulates cancer cells to enhance their amino acid metabolism as a survival mechanism, thereby contributing to cisplatin resistance.

RNA‐seq was performed to clarify the detailed mechanisms through which LAT1‐mediated amino acid metabolism and mTOR‐regulated autophagy contribute to cisplatin resistance. Our analysis identified the *SH3PXD2A* gene as being associated with the mTOR pathway and autophagy [27], suggesting its potential involvement in cisplatin resistance in ESCC. Several studies have conducted RNA‐seq focusing on cisplatin resistance in ESCC, and some have suggested the potential involvement of the mTOR pathway in cisplatin resistance [46]. However, no studies have addressed the potential involvement of *SH3PXD2A* in cisplatin resistance via its association with autophagy. SH3PXD2A expression is reportedly associated with prognosis in ESCC [47]. While the involvement of SH3PXD2A in cisplatin resistance has not been reported in ESCC, it has been associated with cisplatin resistance in non‐small cell lung cancer [48]. Therefore, in this study, we focused on SH3PXD2A. Based on the results obtained thus far, it is suggested that the combination of amino acid supply through LAT1 and autophagy via the mTOR pathway contributes to cisplatin resistance. However, the role of SH3PXD2A in the cisplatin‐resistant cells requires further investigation.

To evaluate the tumor‐suppressive effects of LAT1 inhibition, we conducted a cell proliferation assay using JPH203, a selective inhibitor of LAT1. The tumor‐suppressive effects of JPH203 have been reported in various cancer types, but there are few reports regarding its effects on esophageal cancer [7, 20, 21]. No prior research has investigated the impact of JPH203 on esophageal cancer concerning cisplatin resistance; therefore, we focused on this unexplored aspect. In this study, we classified ESCC cell lines based on their degree of cisplatin resistance and examined the effects of JPH203 on each cell line. JPH203 had a greater effect on cisplatin‐resistant ESCC cell lines, suggesting that LAT1 inhibition may be more effective in cisplatin‐resistant cells. Based on the findings of this study, the combination of JPH203 with cisplatin in a doublet therapy regimen could potentially enhance the efficacy of NAC. Although the safety of JPH203 has been demonstrated in clinical trials, many aspects of its overall safety profile remain unclear. Given that LAT1 is primarily expressed in cancer cells and that JPH203 selectively inhibits LAT1, JPH203 offers the potential for reduced side effects compared to conventional triplet therapy. The expression of LAT1 can be confirmed using molecular imaging with a positron emission tomography scan using ^18^F‐FET, enabling the selection of therapies based on its expression. Further studies are required to confirm these findings.

This study had several limitations. It focused only on surgical specimens obtained after NAC and did not include the evaluation of biopsy specimens obtained prior to NAC as the necessary sample quantity for IHC could not be prepared. Therefore, further studies, including biopsy specimens, are warranted for clinicopathological analysis. However, we demonstrated a significant correlation between high LAT1 expression and NAC efficacy. In addition, the LAT1 inhibitor JPH203 demonstrated the potential to suppress the growth of cisplatin‐resistant ESCC cells. Therefore, the LAT1 expression status could be valuable as both a predictor of prognosis and a treatment target in patients with cisplatin‐resistant esophageal cancer.

In conclusion, we revealed that LAT1 status in patients with ESCC who underwent NAC was significantly associated not only with the clinical outcome but also with the efficacy of NAC. Moreover, we demonstrated that LAT1 may be involved in cisplatin resistance in patients with ESCC. Implementation of a therapeutic strategy that targets LAT1‐mediated amino acid metabolism and the surrounding tumor microenvironment in patients who do not respond to cisplatin could potentially enhance the overall survival rates.

## Author Contributions

**Takeru Mozumi:** writing – original draft, data curation, methodology. **Narumi Harada‐Shoji:** methodology, writing – review and editing, data curation, supervision, project administration, funding acquisition. **Yohei Ozawa:** methodology, writing – review and editing, supervision. **Yuto Yamazaki:** writing – review and editing, supervision, methodology. **Ryoyu Niikuni:** writing – review and editing, methodology, data curation. **Kentaro Imai:** writing – review and editing, methodology, data curation. **Yusuke Taniyama:** writing – review and editing. **Chiaki Sato:** writing – review and editing. **Hiroshi Okamoto:** writing – review and editing. **Hirotaka Ishida:** writing – review and editing. **Atsushi Kunimitsu:** writing – review and editing. **Iku Sasaki‐Higashimoto:** writing – review and editing. **Chisa Kobayashi:** writing – review and editing. **Shozo Furumoto:** writing – review and editing. **Takaaki Abe:** writing – review and editing, supervision, data curation. **Takashi Suzuki:** methodology, writing – review and editing, supervision, data curation. **Takashi Kamei:** project administration, writing – review and editing, supervision.

## Ethics Statement

This study was approved by the Institutional Review Board and Ethics Committee of Tohoku University Hospital (no. 2022–1‐1006).

## Consent

Informed consent was obtained from all the participants via an opt‐out procedure. Those who declined to participate were excluded.

## Conflicts of Interest

The authors declare no conflicts of interest.

## Supporting information


**Data S1:** cam471234‐sup‐0001‐DataS1.pdf.

## Data Availability

The datasets used and/or analyzed during the current study are available from the corresponding author on reasonable request.
