# Supplementary figures and images for: A Novel Approach for Cisplatin‐Resistant Esophageal Squamous Cell Carcinoma via Amino Acid Transporter LAT1 Inhibition
Source: Cancer Med. 2025 Sep 9;14(17):e71234. doi: 10.1002/cam4.71234 (PMC12418084; doi:10.1002/cam4.71234)

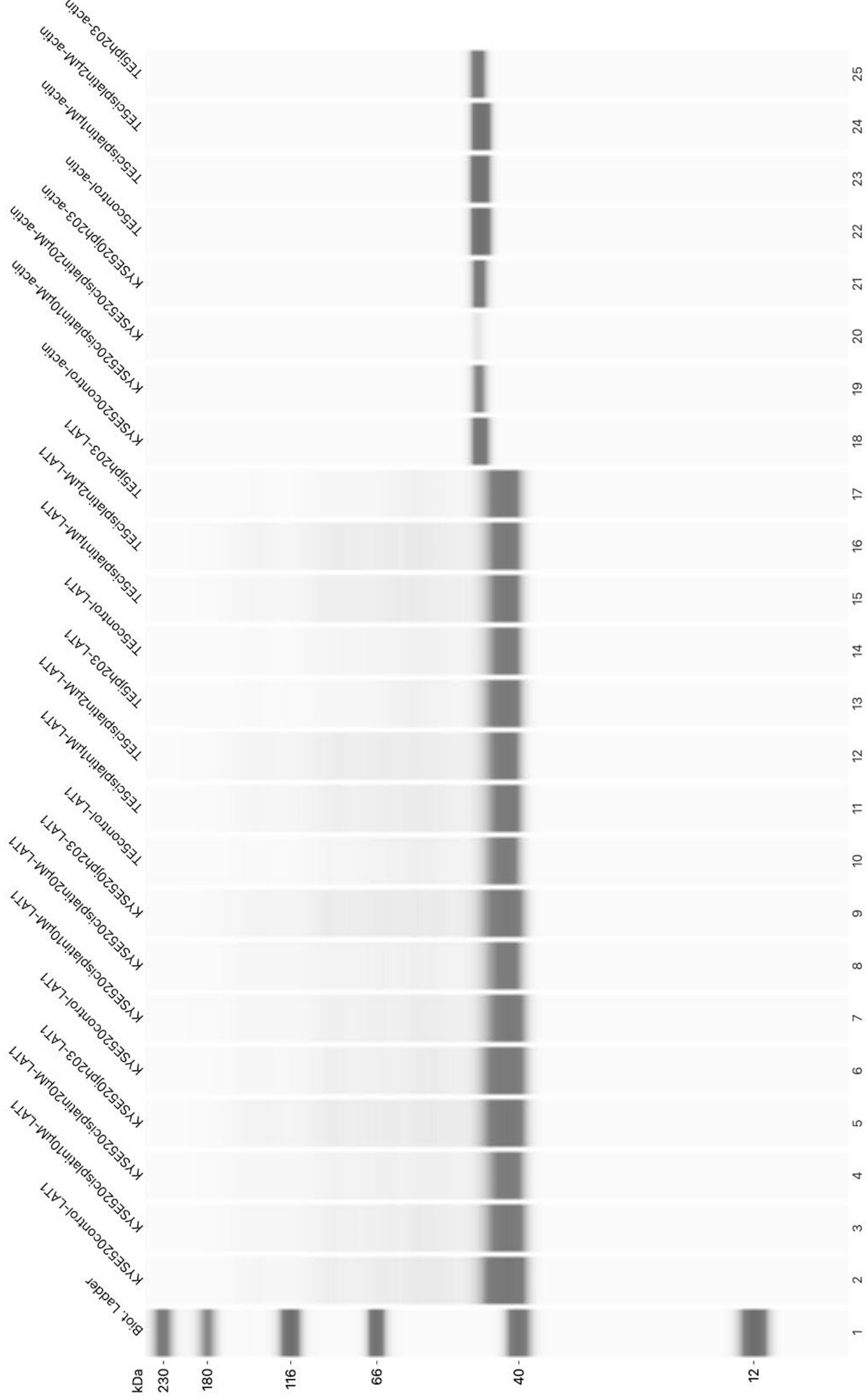

Supplement: Supplementary file 1 — Data S1: cam471234‐sup‐0001‐DataS1.pdf. [file CAM4-14-e71234-s001.pdf]
